# Gut decisions based on the liver: prediction of colorectal neoplasia using AI-based liver analysis of routine CT scans

**DOI:** 10.3389/fonc.2026.1842743

**Published:** 2026-06-03

**Authors:** Anna Hinterberger, Jonas Bohn, Darya Trofimova, Nicolas Knabe, Julia Dettling, Tobias Norajitra, Fabian Isensee, Johannes Betge, Stefan O. Schönberg, Dominik Nörenberg, Sergio Grosu, Sonja Loges, Ralf Floca, Jakob Nikolas Kather, Klaus Maier-Hein, Freba Grawe

**Affiliations:** 1 German Cancer Research Center (DKFZ) Hector Cancer Institute at the University Medical Center Mannheim, Mannheim, Germany; 2 Junior Clinical Cooperation Unit Translational Molecular Imaging in Oncologic Therapy Monitoring (E310), German Cancer Research Center, Heidelberg, Germany; 3 Institute for Diagnostic and Interventional Radiology, Technical University of Munich (TUM) School of Medicine and Health, Technical University of Munich (TUM) University Hospital Kinikum Rechts der Isar, Technical University of Munich, Munich, Germany; 4 Division of Medical Image Computing, German Cancer Research Center (DKFZ), Heidelberg, Germany; 5 Translational Lung Research Center (TLRC), Member of the German Center for Lung Research (DZL), Heidelberg, Germany; 6 Faculty of Biosciences, Heidelberg University, Heidelberg, Germany; 7 The National Center for Tumor Diseases (NCT Heidelberg), Heidelberg, Germany; 8 Helmholtz Imaging, Heidelberg, Germany; 9 Department of Radiology and Nuclear Medicine, University Medical Center Mannheim, Heidelberg University, Mannheim, Germany; 10 Pattern Analysis and Learning Group, Heidelberg University Hospital, Heidelberg, Germany; 11 Department of Medicine II, University Medical Center Mannheim, Medical Faculty Mannheim, Mannheim, Germany; 12 Junior Clinical Cooperation Unit Translational Gastrointestinal Oncology and Preclinical Models, German Cancer Research Center, Heidelberg, Germany; 13 German Cancer Consortium, DKTK, Heidelberg, Germany; 14 Department of Radiology, University Hospital, Ludwig-Maximilians-University (LMU) Munich, Munich, Germany; 15 Division of Personalized Medical Oncology (A420), German Cancer Research Center (DKFZ), Heidelberg, Germany; 16 Department of Personalized Oncology, University Hospital Mannheim, Medical Faculty Mannheim, University of Heidelberg, Mannheim, Germany; 17 Else Kroener Fresenius Center for Digital Health, Faculty of Medicine and University Hospital Carl Gustav Carus, Dresden University of Technology (TUD) Dresden University of Technology, Dresden, Dresden, Germany; 18 Department of Medicine I, University Hospital Dresden, Dresden, Germany; 19 Medical Oncology, National Center for Tumor Diseases (NCT), University Hospital Heidelberg, Heidelberg, Germany; 20 Faculty of Medicine, University of Heidelberg, Heidelberg, Germany; 21 Faculty of Mathematics and Computer Science, Heidelberg University, Heidelberg, Germany

**Keywords:** colorectal neoplasia, CRC-screening, gut-liver-axis, prevention, RPTK

## Abstract

**Introduction:**

Non-invasive colorectal cancer (CRC) screening offers an important opportunity to increase colonoscopy participation and reduce mortality. This study evaluates the potential of the gut–liver axis to predict colorectal neoplasia using artificial intelligence (AI)-based analysis of the liver in routine CT images as an opportunistic screening approach.

**Methods:**

In this retrospective study, data from 1,997 patients were analyzed, including 1,189 without neoplasia and 808 with colorectal neoplasia (423 adenomas, 385 CRC). Radiomic features were extracted from three-dimensional liver segmentations, and the dataset was split into training (n = 1,397) and test (n = 600) cohorts. Five machine learning models were trained using five-fold cross-validation on the 20 most informative features.

**Results:**

The best-performing radiomics-based XGBoost model achieved a test AUROC of 0.810 (95% CI: 0.767–0.837), outperforming a clinical-only model (AUROC: 0.457). After threshold optimization, sensitivity reached 74.1% and specificity 72.3% for detecting colorectal neoplasia. Subclassification between CRC and adenoma was less accurate (AUROC: 0.674).

**Discussion:**

These findings demonstrate that AI-based liver analysis from routine CT scans can predict colorectal neoplasia, supporting its potential as an accessible adjunct to CRC screening and highlighting the gut–liver axis as a novel biomarker source.

## Introduction

Colorectal cancer (CRC) is largely preventable through the detection and removal of precancerous lesions during screening colonoscopy (1, 2). Despite the availability of organized screening programs, CRC remained the third leading cause of cancer-related mortality rate worldwide in 2022, likely reflecting low screening participation rates (3, 4), which vary considerably, with one German study even reporting rates as low as 20% (5–7). Given the recommended participation rate of 80% to achieve a substantial reduction in CRC mortality (4), current rates are clearly insufficient, underscoring the urgent need to optimize screening strategies and develop novel approaches to increase participation (8, 9).

One potential strategy is to harness existing routinely available computed tomography (CT) imaging data for individual CRC risk prediction. However, the colon is difficult to evaluate in CT examinations, which were not performed following a dedicated CT colonography protocol (10). In contrast, the liver is an ideal target organ for opportunistic analysis in CT due to the feasibility of robust automated segmentation and analysis. Owing to its central role in metabolic processes, the liver has shown to have predictive potential for chronic disease risk. For instance, imaging features of the liver have previously been shown to predict cardiovascular conditions (11). In the context of CRC, the interplay within the gut-liver axis represents a critical mechanistic link: disrupted bile acid metabolism in conditions such as primary sclerosing cholangitis (PSC) and metabolic dysfunction-associated steatotic liver disease (MASLD), as well as inflammatory mediators transported via the portal system in patients with inflammatory bowel disease (IBD), contribute to chronic hepatic diseases and colonic neoplasia such as CRC (12, 13). Moreover, shared risk factors, such as obesity, elevated body mass index, diabetes and metabolic syndrome further link chronic liver diseases and colorectal neoplasia (14).

Building on these pathophysiological foundations, machine learning (ML) models provide a powerful approach to extract hepatic features from medical imaging as potential biomarkers for CRC risk stratification. By enabling the quantification of biomedical information, ML methods facilitate the integration of various imaging and clinical data into risk prediction algorithms, an approach that has already been successfully applied to predict e.g. cardiovascular risk based on liver magnetic resonance imaging (MRI) data (11, 15).

Leveraging the gut-liver axis, this proof-of-concept study suggests that imaging features of the liver, extracted from routine CT, may serve as non-invasive biomarkers of colorectal neoplasia as an additional opportunistic, efficient screening method besides established CRC screening methods. Importantly, this approach could make use of CT examinations performed for other clinical indications, thereby broadening the potential for early risk assessment beyond dedicated screening settings. Ultimately, this study seeks to establish a first step toward accessible, low-threshold CRC screening strategies without imposing additional time or cost burdens on patients or clinicians.

## Methods

### Study population

**Figure 1:**
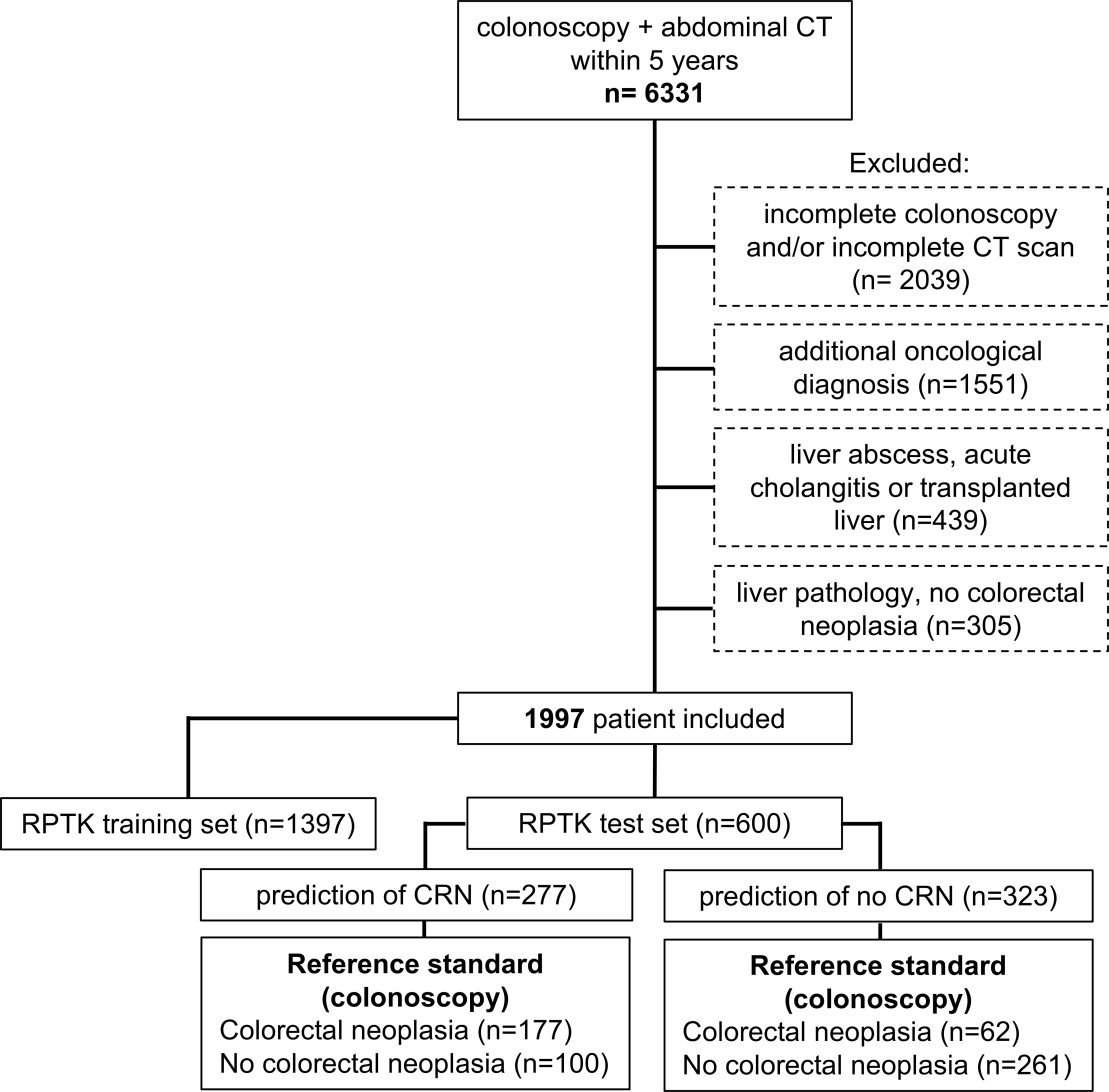
STARD flow-chart of patients’ cohort, the training set and test set. Additional solid tumors refer to all oncological diagnoses of solid cancers that have a known tendency of metastasizing into the liver (such as breast cancer) and solid cancer with the liver being the known primary region (e.g. hepatocellular carcinoma). Colorectal neoplasia was diagnosed and characterized during colonoscopy in clinical routine. Liver diagnoses were extracted from the internal clinical reporting system and are based on imaging and/or laboratory diagnosis. Incomplete colonoscopy is defined as colonoscopy which did not reach the coecum. RPTK, Radiomics Processing ToolKit; CRN, colorectal neoplasia..

This retrospective study was approved by the local ethics committee (EK II 2023-887-AF 11). All patients who underwent colonoscopy and had a CT-scan of the abdomen were included in this study. Patient data from colonoscopies performed in clinical routine between 1 January 2013 and 29 December 2023 at a single center were reviewed (n=12, 355) and cross-checked with the local imaging database to determine whether an abdominal CT scan was performed within five years before or after colonoscopy, resulting in 6, 331 patients. After applying further exclusion criteria (see **Figure 1**), a total of 1, 997 patients were included in the final analysis. The Checklist for Artificial Intelligence in Medical Imaging (CLEAR) and Standards for Reporting of Diagnostic Accuracy (STARD) were applied to ensure transparency, reproducibility, and high-quality reporting (16, 17).

### Colonoscopy and liver diagnosis data

The ground truth for colorectal neoplasia was defined based on histopathological diagnoses and colonoscopy findings, systematically extracted by trained medical study personnel. For liver pathologies, diagnoses were derived from general clinical reports and liver sonography reports, with data extraction performed by the same trained personnel. Patients were classified according to their most advanced finding at colonoscopy as follows: i) CRC, ii) adenoma (further subclassified into advanced and non-advanced adenomas), or iii) no colorectal neoplasia. Advanced adenomas were defined by at least one of the following: size > 1 cm, tubulovillous or villous architecture, or high-grade dysplasia (**Table 1**).

**Table 1:** Patients’ characteristics.

Patients’ characteristics	n (%)
men	1138 (57%)
women	859 (43%)
mean age at CT scan (years)	64
mean time between colonoscopy and CT (months)	7
CT prior colonoscopy	1650 (83%)
IBD	158 (8%)
CHE	207 (1%)
colorectal neoplasia	808 (40%)
CRC	385 (48%)
adenoma	423 (52%)
advanced adenoma	141 (33%)
right sided neoplasia	336 (41%)
no liver pathology	643 (79%)
diagnosed liver pathology	165 (21%)
MASLD	74 (44%)
toxic and alcoholic liver diseases	15 (8%)
cirrhosis	76 (47%)
no colorectal neoplasia or liver pathology	1189 (60%)

IBD, inflammatory bowel disease; CHE, cholecystectomy; CRC, colorectal cancer; MASLD, metabolic associated steatotic liver disease.

Liver diagnoses were extracted from the local hospital information system (SAP Deutschland SE & Co. KG, Walldorf, Germany) based on the 10th revision of the International Statistical Classification of Diseases and Related Health Problems (ICD-10) codes K70.- to K77.-. Diagnoses were based on imaging and/or laboratory results. Patients with diagnosis of solid liver tumor, liver abscess or metastases in the liver were excluded as detailed in **Figure 1**.

### Image acquisition and segmentation

All included CT scans came from a single center using ten different CT scanners (see **Supplementary Table 1** for further details). Scans were selected if they were acquired with contrast agent (Imeron**^®^**350, Bracco Imaging Deutschland GmbH, Konstanz, Germany) in portal venous phase (70 seconds post-injection). This protocol was chosen as it is the most frequently used abdominal CT protocol in clinical routine across a wide range of indications, which is essential for ensuring broad applicability of the algorithm trained on these scans.

Liver segmentations were generated by applying MultiTalent, a model built upon the nnU-Net framework, to the 3D CT scans (18, 19). MultiTalent was trained on 13 public abdominal CT datasets (1, 477 images, 47 classes). For inference, we applied the pretrained model in evaluation mode, using only its liver-specific sigmoid head to generate binary liver masks for downstream analysis without additional fine-tuning. A random subset of the liver segmentations were manually inspected in order to confirm anatomical plausibility.

### Feature engineering

We used the Radiomics Processing Toolkit (RPTK) (20), to generate reproducible standardized radiomics features, achieving state-of-the-art quality for reproducibility and transparency according to the CLEAR guidelines (17). The RPTK tool used for radiomics experiments in this study is publicly available on GitHub (https://github.com/MIC-DKFZ/RPTK). All images and corresponding segmentations were resampled to an isotropic voxel spacing of 1 mm³. Image resampling was performed using third-order B-spline interpolation, while segmentations were resampled using nearest-neighbor interpolation to preserve label integrity. To reduce segmentation artifacts, connected component analysis was applied, and only the largest connected component, corresponding to the main liver volume, was retained for subsequent analysis. Disconnected mask components were removed before radiomic feature extraction and therefore did not contribute to feature computation, model training, or prediction. Radiomic features were extracted without applying any additional image transformations or mask perturbations. Intensity discretization was performed using a fixed bin width of 25 prior to feature computation. All resampling procedures were implemented using the SimpleITK library (21). A total of 227 radiomic features - covering eight different feature classes - were extracted using PyRadiomics (22) (**Supplementary Figure 1**), and subsequently normalized using z-score normalization.

**Figure 2:**
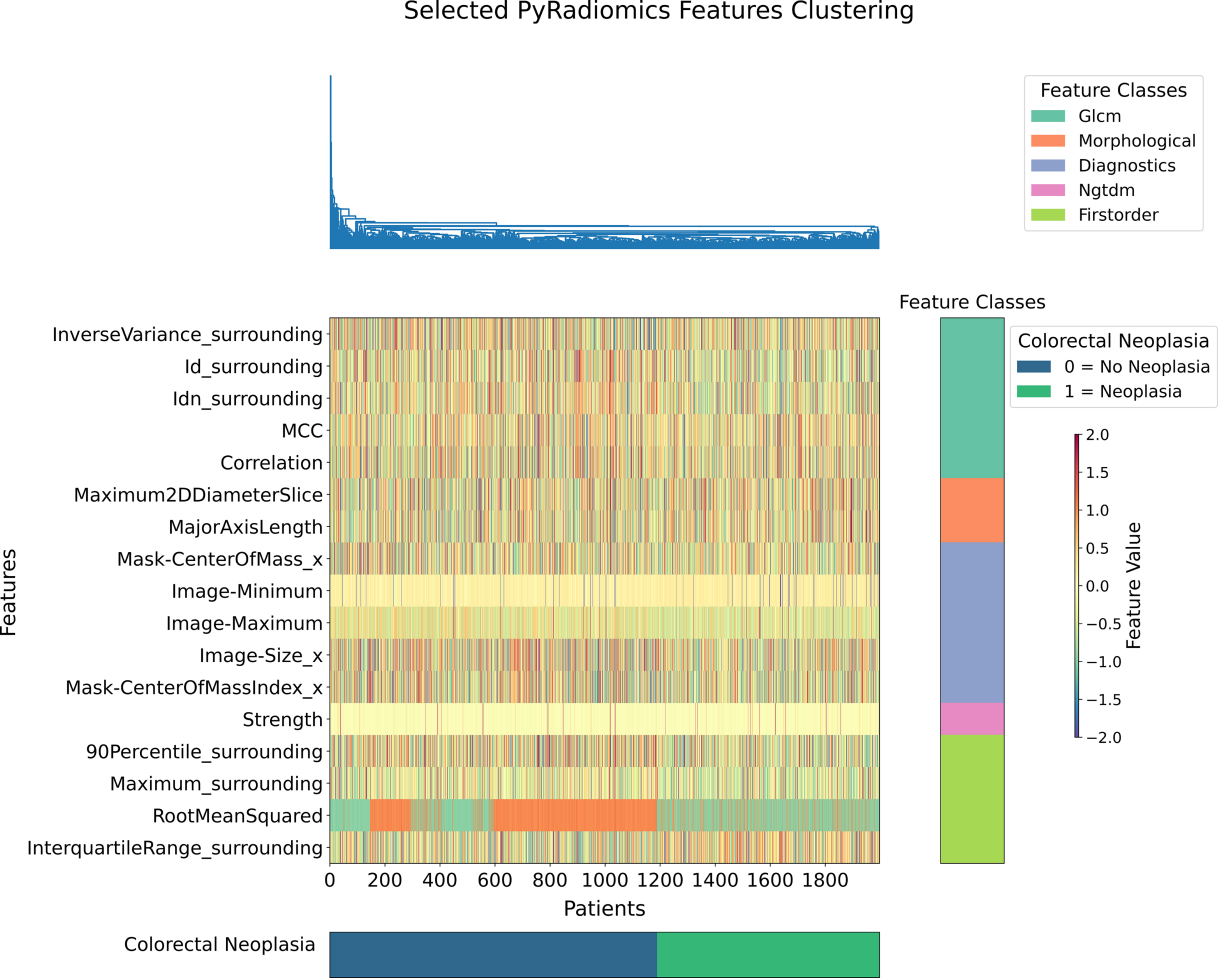
Stratification of selected radiomics features from PyRadiomics for colorectal neoplasia based on the features extracted from the liver with a best stratifying root mean squared and 90th percentile. No clinical parameter was determined to be relevant (Id: Inverse Difference, Idn, Inverse Difference Normalized; MCC, Maximal Correlation Coefficient).

In addition to the liver, the liver-surrounding region was analyzed, and radiomic features were extracted and integrated into the feature space. Afterwards, correlation filtering with a pearson correlation coefficient threshold of r=0.9 and a variation filtering with a threshold of r=0.1 was performed. Feature selection was performed using sequential selection by using a random forest classifier, resulting in a final set of 18 features that were used to predict colorectal neoplasia (**Figure 2**). The final selected features, including the corresponding PyRadiomics feature class and individual feature name, are shown in **Figure 2**. These radiomic features represent quantitative CT-derived intensity, shape, and texture descriptors as defined by PyRadiomics. These features were used to predict colorectal neoplasia based on liver-related imaging features. No feature selection was performed on the test set.

Clinical features (chronic liver disease, colon pathology, age, sex, cholecystectomy and inflammatory bowel disease) were also included to identify relevant clinical risk factors. There was no significant correlation with any clinical paramter and the presence of colorectal neoplasia (**Supplementary Figure 2**). Therefore, none of the clinical features were retained in the final model based on missing performance gain during the iterative selection process and prediction of colorectal neoplasia was based solely on radiomic features. The heatmap (**Figure 2**) illustrates clear differentiation patterns between patients with and without colorectal neoplasia based on specific radiomics-derived texture and morphological features. Feature clustering revealed that classes such as grey level co-occurrence matrix and first-order statistics contributed most prominently to this stratification.

### Model training

The dataset was randomly split into training and test set using sklearn (23) (**Figure 1**). Five model types - Random Forest, TabNet, Light Gradient Boost Model (LGBM), Extreme Gradient Boost Model (XGBoost), Support Vector Machine (SVM) - were trained, cross-validated (five-fold), and optimized on the training set, which included 1397 randomly selected cases. The best optimized ensembled model were reported and applied to the held-out test set (including 600. Cases). SMOTE (Synthetic Minority Over-sampling Technique) was applied to address class imbalance in the training dataset for the sub-classifications of CRC vs. adenoma and CRC/adenoma vs. no colorectal neoplasia. After training the five models on 5 different folds, the fold-specific models were ensembled. The best model type was selected based on its averaged cross-validation AUROC.

### Statistical analysis

**Figure 3:**
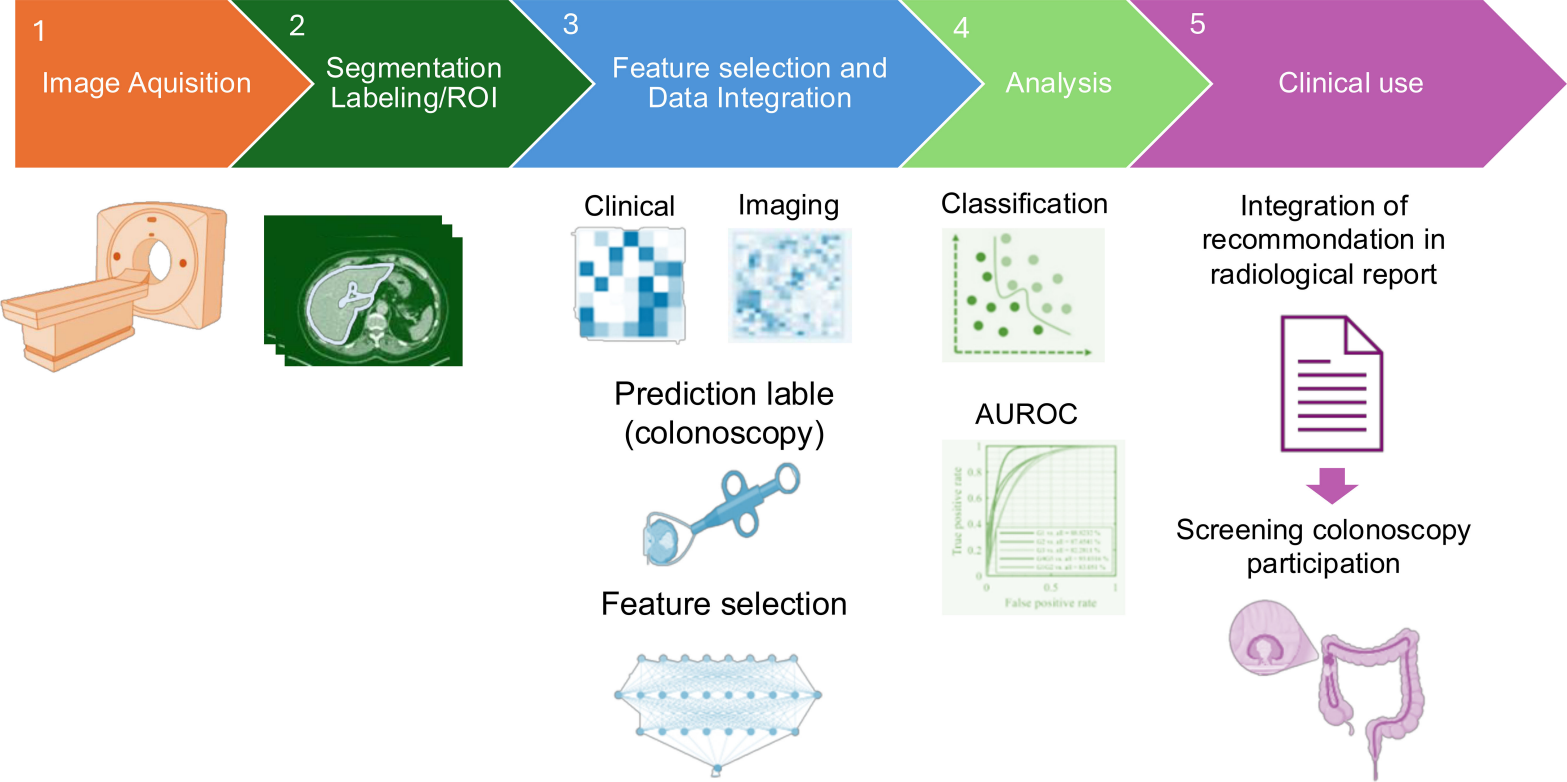
Data processing flow-chart. The workflow of LIRA includes five continuous parts: (1) Image acquisition of abdominal CT scans. (2) Liver segmentation, with automatic segmentation methods. (3) Model building and data integration of colonoscopy diagnosis functioning as prediction target. (4) Statistical analysis of model performance. (5) clinical use. AUROC, area under the receiver operating curve. Own illustration.

Model performance was evaluated using the area under the receiver operating characteristic curve (AUROC), sensitivity and specificity. The optimal classification threshold was derived by maximizing the Youden Index (sensitivity + specificity − 1) on the training dataset. This threshold was subsequently applied to the test dataset to assess model performance in an unbiased manner and to prevent information leakage. SMOTE was applied only during model training and was not applied to the held-out test set. The Youden threshold selected on the training data was fixed before test-set evaluation and applied unchanged to the original, non-synthetic test cohort. Correlations between clinical parameters were assessed using the Pearson correlation coefficient. Descriptive values are presented as means with standard deviations (SD) or as percentages (%), as appropriate. Statistical analyses were performed using RStudio (PositPBC, Boston, Massachusetts, US, Version 2024.12.1). For the confusion matrix evaluation and ROC analysis, sklearn (version 1.5.0) was used (23). A summary of the data processing approach is given in **Figure 3**.

## Results

### Model predicts colorectal neoplasia based on radiomic features extracted from CT-scans of the liver

Multiple ML models were applied to predict the presence of colorectal neoplasia based on radiomics extracted from the liver on CT. After training on the selected radiomic features from PyRadiomics with RPTK, the best-performing model was the XGBoost model (Validation AUROC: 0.832 ± 0.013; Test AUROC: 0.810 [95% CI: 0.767 - 0.837] (**Supplementary Figure 3**). In contrast, a model based solely on clinical data performed significantly worse, with the best clinical-only XGBoost model yielding near-random classification performance (Validation AUROC: 0.547 ± 0.018; Test AUROC: 0.457 [95% CI: 0.411-0.506] (**Supplementary Figure 4**).

**Figure 4:**
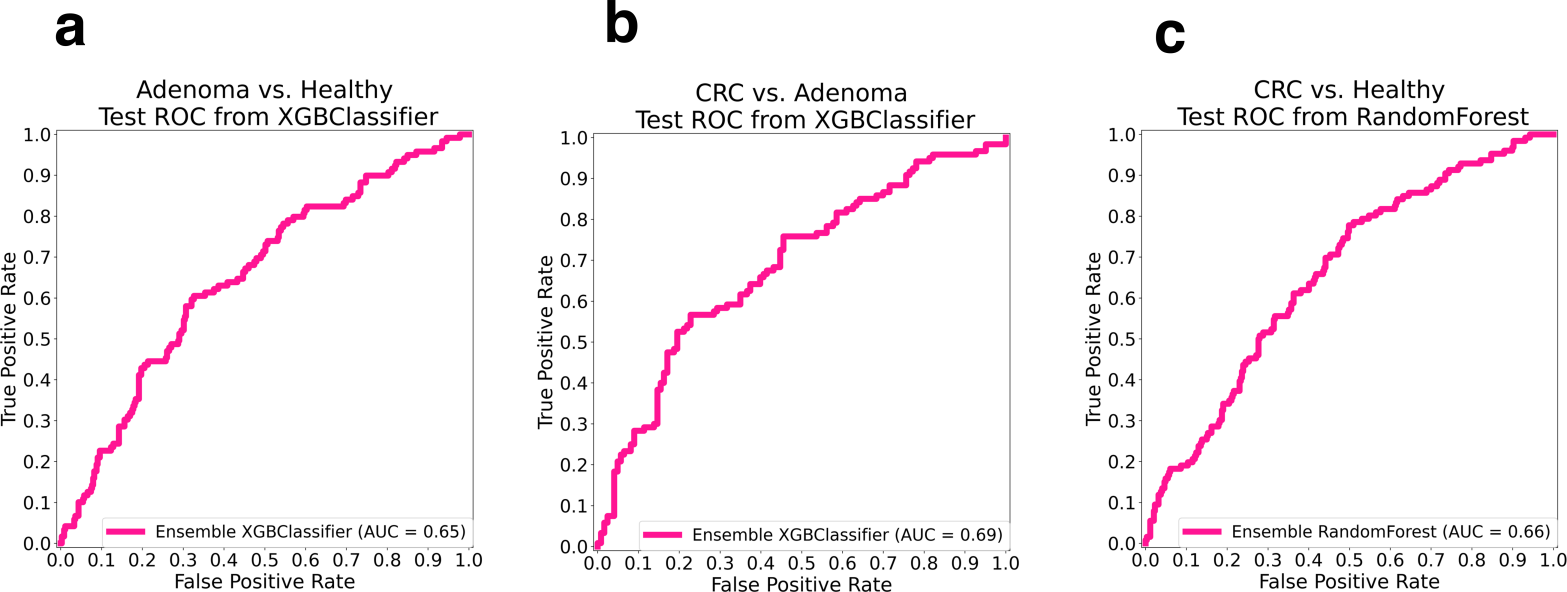
**(a)** ROC curve ensemble on the test set of the best-performed model (XGBoost Classifier) for discrimination of CRC and adenoma. **(b)** ROC curve ensemble on the test set of the best-performed model (Random Forest Classifier) for discrimination of CRC and healthy patients. **(c)** ROC curve ensemble on the test set of the best-performed model (XGBoost Classifier) for discrimination of adenoma and healthy patients..

Model selection was based on the highest validation AUROC to ensure robust generalizability (see **Supplementary Figures 3**, **S4**). **Figures 4a, b** display the corresponding ROC curves for the cross-validation training and the ensembled model applied to the test set.

### Models sensitivity and specificity for classification of colorectal neoplasia vs. no colorectal neoplasia

**Figure 5:**
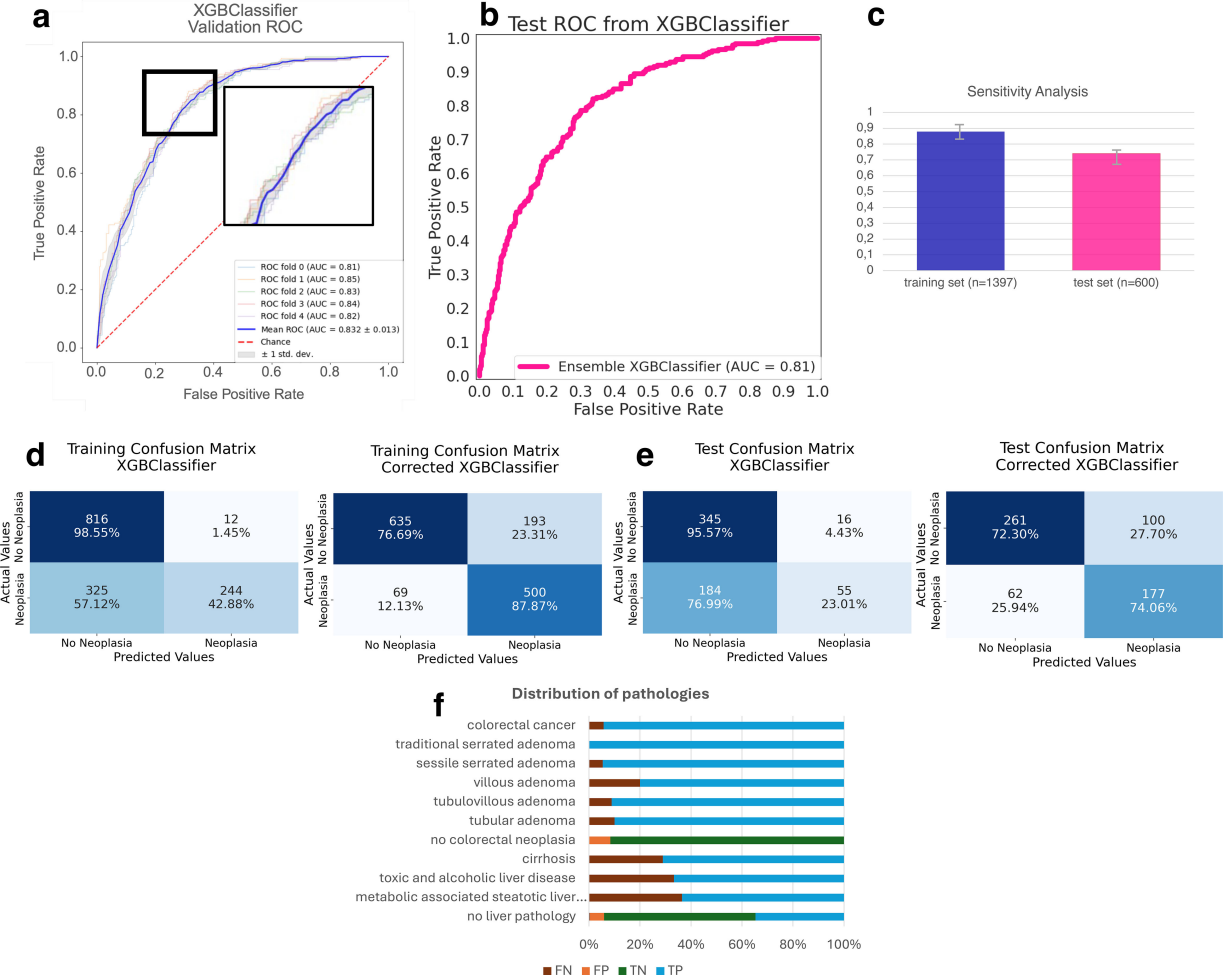
**(a)** ROC Curves of the 5-fold validation set of the best-performed model (XGBoost Classifier) for colorectal neoplasia prediction. **(b)** ROC curve ensemble on the test set of the best-performed model (XGBoost Classifier) for colorectal neoplasia prediction. **(c)** sensitivity of XGBoost Classifier for colorectal neoplasia prediction in the trainings set (n=1, 397) and test set (n=600). The error bars denote the two-sided 95% CI. **(d)** confusion matrices of the best model on the training set before (left) and after (right) Youden index correction for optimization of threshold-based matrices. **(e)** confusion matrices of the best model on the test set before (left) and after (right) Youden index correction for optimization of threshold-based matrices (Label: 0= no colorectal neoplasia, 1= colorectal neoplasia). **(f)** Disruption of liver and colon pathologies within the test set, subclassified in true positive (TP), false negative (FN), false positive (FP) and true negative (TN) results for the prediction of colorectal neoplasia of the XGBoost Classifier.

The evaluation of the sensitivity and specificity analysis in the context of classifying colorectal neoplasia vs. the absence of colorectal neoplasia demonstrated improved performance of the XGBoost model following threshold optimization via the Youden Index. As shown in **Figure 5e**, the initial confusion matrix (left) reflected a relatively high specificity (95.6%) but a markedly lower sensitivity (23.0%) on the test set. After weighting of sensitivity and specificity and post-adjustment of the classification threshold based on the Youden J statistic (J = 0.667), the best performing corrected model (right) achieved a more balanced performance, with a sensitivity of 74.1% and a specificity of 72.3%. The model also showed generalizability, with training set sensitivity and specificity of 87.9% and 76.7% (**Figures 5c, d**), respectively, and corresponding test set values of 74.1% and 72.3% (**Figures 5c, e**). When further analyzing the true positive and false negative predicted cohort, we found that only 14.9% ± 36.0% of the patients had liver diseases and 5.7% ± 34.0% in the cohort of false negatives had diagnosed liver diseases (**Figure 5f**).

### Models differentiates between CRC and adenoma

To further evaluate the model’s capacity for sub-classification within colorectal neoplasia, we assessed its performance in differentiating between adenoma and CRC using both radiomic and clinical features. The best-performing approach utilized radiomics-derived features in combination with clinical data, achieving an AUROC of 0.671 ± 0.018 on the validation set and AUROC 0.674 [95% CI: 0.606 - 0.741] on the test set using an XGBoost classifier (**Figure 4a**). This indicates moderate discriminative ability between adenoma and CRC lesions (**Supplementary Figure 5**). Further classification of adenoma, or CRC against healthy patients resulted in validation AUROC: 0.913 ± 0.014 and test AUROC: 0.659 [95% CI: 0.606-0.712] with Random Forest Classifier for CRC (**Figure 4b**, **Supplementary Figure 6**) and validation AUROC: 0.873 ± 0.009 and test AUROC: 0.651 [95% CI: 0.592-0.705] with XGBoost for adenoma (**Figure 4c**, **Supplementary Figure 7**).

## Discussion

This study demonstrates that automated AI-based analysis of the liver on contrast-enhanced abdominal CT can non-invasively predict colorectal neoplasia with high accuracy. In a large, real-world cohort of nearly 2, 000 patients who underwent both colonoscopy and abdominal CT, our XGBoost model achieved robust predictive performance (validation AUROC: 0.832 ± 0.013; test AUROC: 0.810 [95% CI: 0.767–0.837]). These results indicate that liver-derived imaging features may reflect hepatic processes associated with colorectal tumorigenesis, highlighting the gut–liver axis as a relevant biomarker source. Consequently, our findings offer a foundation for a new biomechanical opportunity to screen for CRC and support integration of such AI-tools into national screening programs as an adjunct to established methods such as colonoscopy, particularly relevant given persistently low participation rates in CRC screening in Germany and other countries.

The high number of CT scans conducted annually in Germany, estimated at 160 per 1, 000 individuals or approximately 13.3 million scans in 2021 (24), underlines the potential public health impact of such an opportunistic screening approach. For instance, if colorectal neoplasia is predicted using our AI tool (positive test outcome message such as “high probability that colorectal neoplasia might be present” is written for the radiologist), this information could be directly communicated in radiologic reports, thereby prompting timely recommendations for colonoscopy. Moreover, if the AI tool yields a positive result, the radiologist could perform a more detailed evaluation of the colon, where adenomas or small colorectal cancers (CRCs) can easily be overlooked on CT scans. Owing to its opportunistic application, this method does not generate additional costs for the healthcare system and does not impose any further burden or time investment on patients or clinicians, as no time investment or bowel preparation is needed, unlike CT colonography and the tool could run in parallel to image reconstruction e.g. on a connected server infrastructure and. In such a scenario, inference would be performed automatically on each image series immediately after acquisition, with short processing times and overall latencies well below clinically relevant thresholds, contributing to a more structured and standardized documentation of findings, improving consistency and clarity.

This approach also addresses the low participation in traditional screening, by intervening in the clinical workflow where patients are already engaged (25, 26). Moreover, this approach could also help mitigate the under-detection of cancer in patients with lower socioeconomic status, as disparities in access and participation are well documented in screening programs such as those for breast cancer (27). By providing a low-threshold, easily deployable triage tool, our model may facilitate earlier identification of at-risk individuals who might otherwise not engage with established screening pathways.

Our method may also enhance post-screening surveillance. Post-imaging colorectal cancer (PICRC) remains a challenge, with rates of 4–6% (28, 29); our strategy could offer early identification of patients at risk for PICRC in the interval between colonoscopies.

While the association between chronic liver disease and CRC is well known (13, 14), automated colorectal neoplasia prediction based on biomedical features extracted from the liver is not explored in this setting yet. However, comparable approaches, using radiomic features from a surrogate or remote organ have already been validated in other clinical scenarios, for example, using pancreatic volume to predict cancer cachexia in head and neck cancer patients (30), or for the prediction of multiple chronic diseases based on liver features such as cardiovascular events (11), highlighting the potential of utilizing inter-organ crosstalk for cancer screening, therapy monitoring, and advanced risk stratification.

In line with more established screening modalities like FOBT (31), differentiation between advanced neoplastic subtypes remains moderate in our study with an AUROC for adenoma detection of 0.651 [95% CI: 0.592–0.705] and an AUROC for distinguishing adenomas from CRC of 0.674 [95% CI: 0.606–0.741], likely reflecting subtler changes in early adenomatous stages and class imbalance.

Previous studies evaluating clinical risk models reported moderate diagnostic performance, with exemplarily Chen et al. achieving AUROC of 0.75 (32). In our study, clinical data alone showed poor performance (test AUROC: 0.457 [95% CI: 0.411–0.506]) and was substantially inferior to our radiomics-based approach (test AUROC: 0.810 [95% CI: 0.767–0.837]). This finding can potentially be attributed to the limited number of clinical parameters included in the study as well as the very homogeneous age and sex distribution between patients with and without colorectal neoplasia.

Nevertheless, these results illustrate that AI-based liver analysis in routine CT may capture subtle, even subclinical, hepatic changes linked to colorectal neoplasia, as suggested by the finding that only 14.9% of correctly classified positive cases had documented liver disease.

The broader field has already demonstrated high diagnostic accuracy of radiomic-based AI-models for other malignancies such as hepatocellular carcinoma or pancreatic ductal adenocarcinoma, reporting AUROCs above 0.90 (33, 34). Adoption of AI-based CRC screening has been limited, likely due to technical challenges in colon segmentation (35); therefore, our solution focuses on the liver as a surrogate, which may facilitate wider implementation and further validation with direct analysis on segmented colon abnormalities.

While our model’s sensitivity (74.1%) and specificity (72.3%) are encouraging, they remain moderate when considering the requirements for a primary screening tool (36). In general, it is important to emphasize that we do not propose replacing established CRC screening methods, but rather aim to provide a complementary fully automated approach, particularly for patients with existing CT data, where an opportunistic strategy can be applied. To further improve future opportunistic screening approaches, integration of AI-based liver analysis in routine CT with other non-invasive risk factors such as liquid biopsy, and advanced imaging modalities (e.g. MRI or ultrasound-based elastography) as well as inclusion of additional clinical or lifestyle data could be considered. In addition, future validation studies may incorporate SHAP-based feature-attribution analyses to further characterize global and patient-level contributions of individual radiomic features.

Key limitations of our study include its retrospective, single-center design and the heterogeneity of indications for colonoscopy, which may introduce selection bias. The temporal sequence, where some CT scans were performed after adenoma removal, may attenuate detected associations. Moreover, our cohort shows a high prevalence of CRC (48%) compared to the prevalence of adenomas (52%), which reflects the university hospital setting where many patients are referred for carcinoma treatment, while only a small proportion undergo initial screening. As this work represents a proof-of-concept analysis, external validation in larger, prospectively recruited multicenter screening cohorts, which currently underway in our group, is essential before clinical implementation. In addition, as SMOTE may influence score calibration and cutoff selection, future external validation should assess cutoff stability and, if necessary, recalibrate the decision threshold in cohorts with representative disease prevalence.

In summary, AI-based analysis of the liver in routine CT scans represents a promising and widely accessible clinical tool for CRC screening. Embedding this approach into established clinical workflows could enhance screening uptake and facilitate earlier CRC detection and prevention. Ultimately, by exploring the gut-liver axis, this strategy may spark new mechanistic hypotheses that shape the next generation of precision prevention strategies.

## Data Availability

The raw data supporting the conclusions of this article will be made available by the authors, without undue reservation.
