# Supplementary material for: Gut decisions based on the liver: prediction of colorectal neoplasia using AI-based liver analysis of routine CT scans
Source: Front Oncol. 2026 Jun 3;16:1842743. doi: 10.3389/fonc.2026.1842743 (PMC13272017; doi:10.3389/fonc.2026.1842743)
Supplement: Supplementary file 1 [file DataSheet1.docx]

**Supplementary Figures and Tables**

| Included kernels | B30f, B30s, B30, B31f, B40f, Br 36, Br38, Br39, Br40, Bv45, I30, I30s, I30f, I31f, Q30f |
| --- | --- |
| CT scanners used in training set | Biograph 40 (n=48; 3%), Emotion 16 (n=388; 28%), NAEOTOM Alpha (n=20; 1%), Sensation 64 (n=273; 20%), SOMATOM Definition (n=8; 1%), SOMATOM Definition AS+ (n=271; 19%), SOMATOM Definition Flash (n=295; 21%), SOMATOM Force (n=32; 2%), SOMATOM go.Up (n=63; 5%) |
| CT scanners used in test set | Biograph 40 (n=19; 3%), Emotion 16 (n=147; 25%), NAEOTOM Alpha (n=90; 3%), Sensation 64 (n=115; 19%), SOMATOM Definition (n=4; 1%), SOMATOM Definition AS+ (n=119; 20%), SOMATOM Definition Flash (n=130; 22%), SOMATOM Force (n=13; 2%), SOMATOM go.Up (n=32; 5%); Volume Zoom (n=4; 1%) |
| Slice thickness [mm] | Mean=4.6 (SD=0.80) |
| Number of slices | Mean=111,27 (SD=53,26) |
| ROI size [Voxels] | Mean=602858.49 (SD=441908) |
| Number of connected components | Mean=1.43 (SD=2.14) |
| Number of bins (with bin width of 25) | Mean=12.89 (SD=6.53) |
| Number of grey values in ROI | Mean=251.42 (SD=56.92) |
| Mean grey value in ROI | Mean=91.15 (SD=22.22) |
| Surface wavelet | Mean=44.84 (SD=11.08) |
| Mesh curvature | Mean=-0.017 (SD=0.008) |
| Fractal dimension 3D | Mean=2.44 (SD=0.09) |

*Table S1. Detailed information of CT values.*


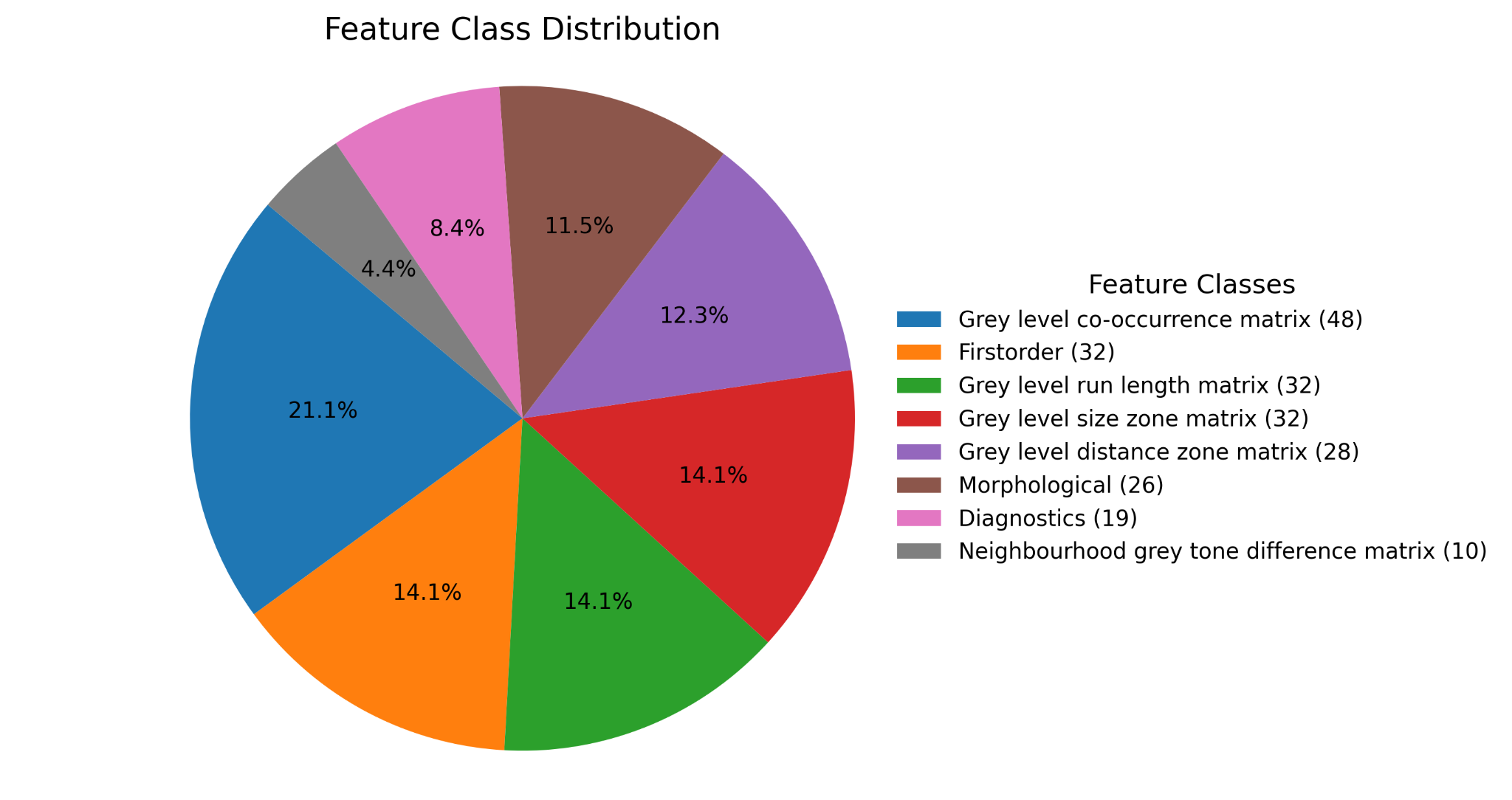


*Figure S1. Distribution of radiomics feature classes in the raw feature extraction.*


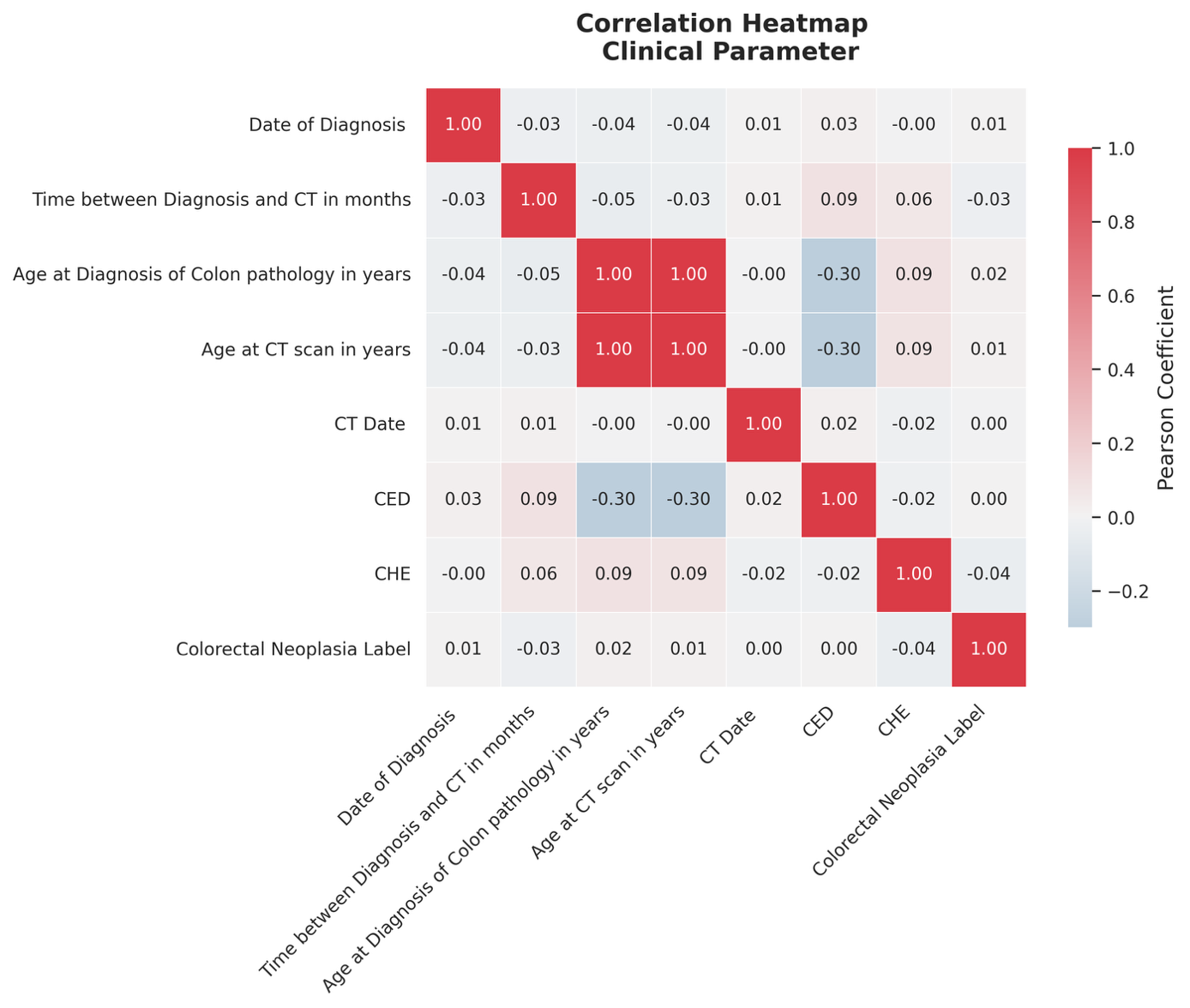


*Figure S2. Clinical feature correlation map including Pearson correlation coefficient to the colorectal neoplasia.*


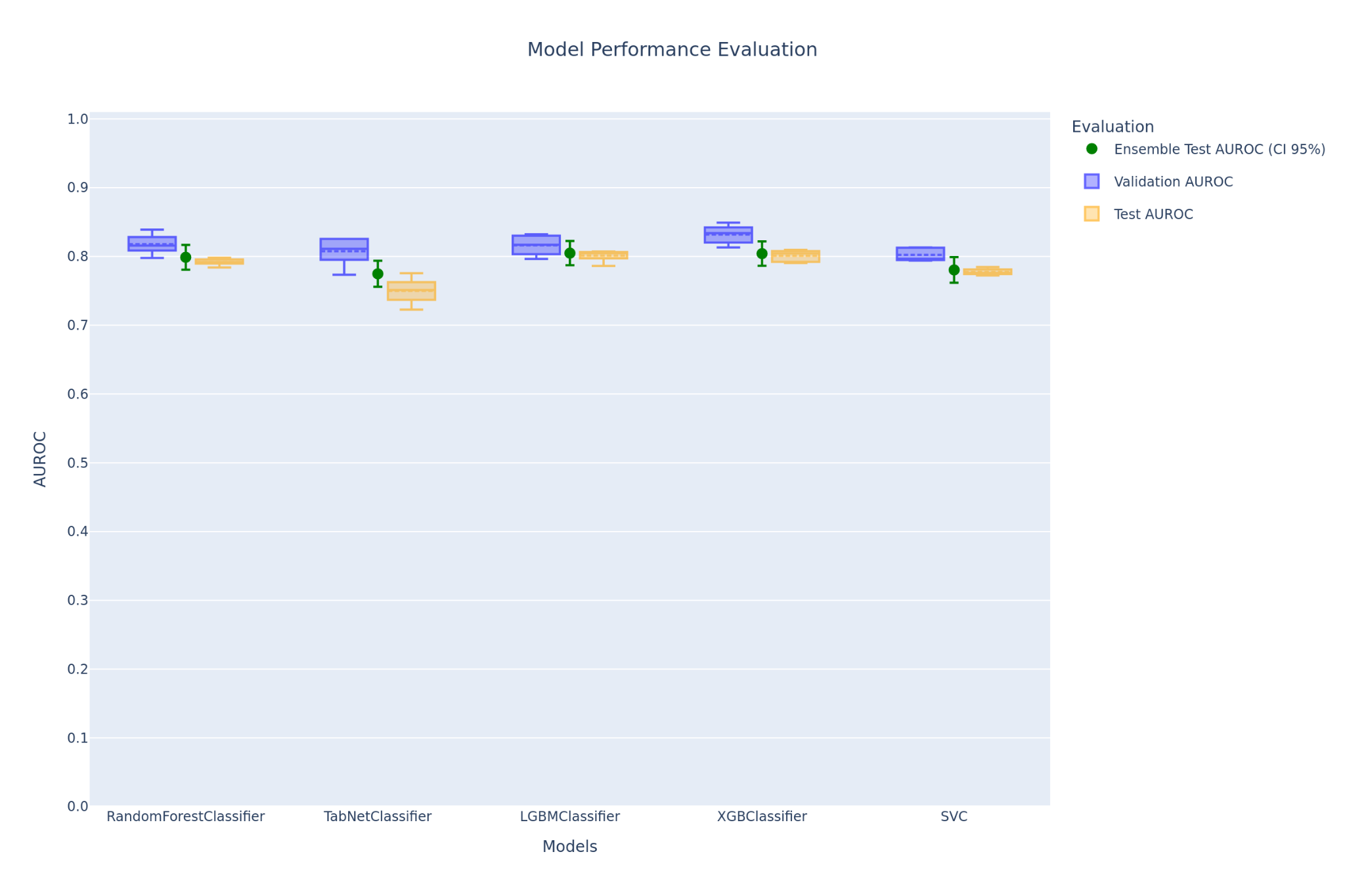


*Figure S3. Summary of AUROC performance of applied models on PyRadiomics features for colorectal neoplasia prediction using the RPTK framework.*

*t
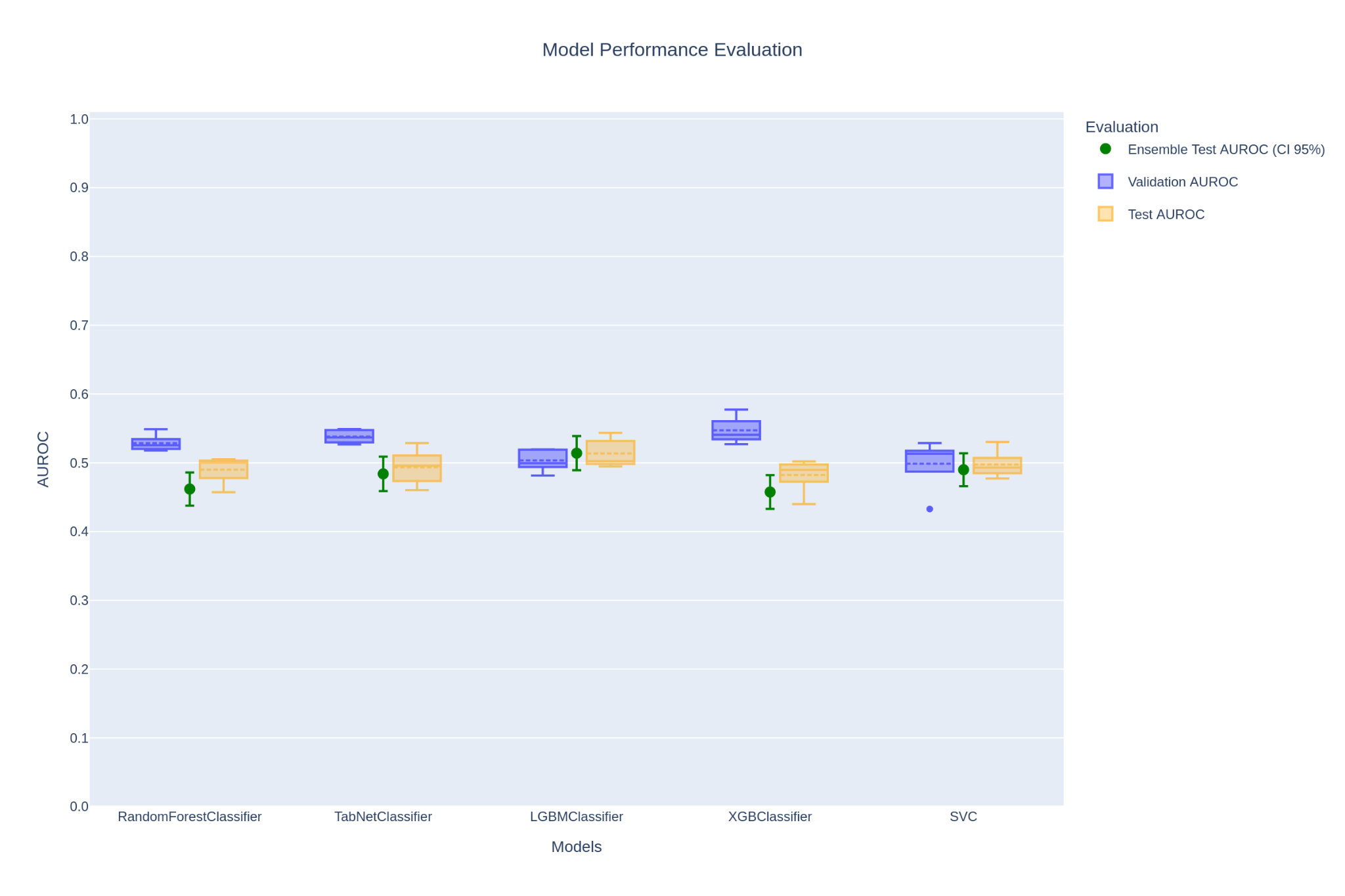
*

*Figure S4. Summary of AUROC performance of applied models on clinical features for colorectal neoplasia prediction using*


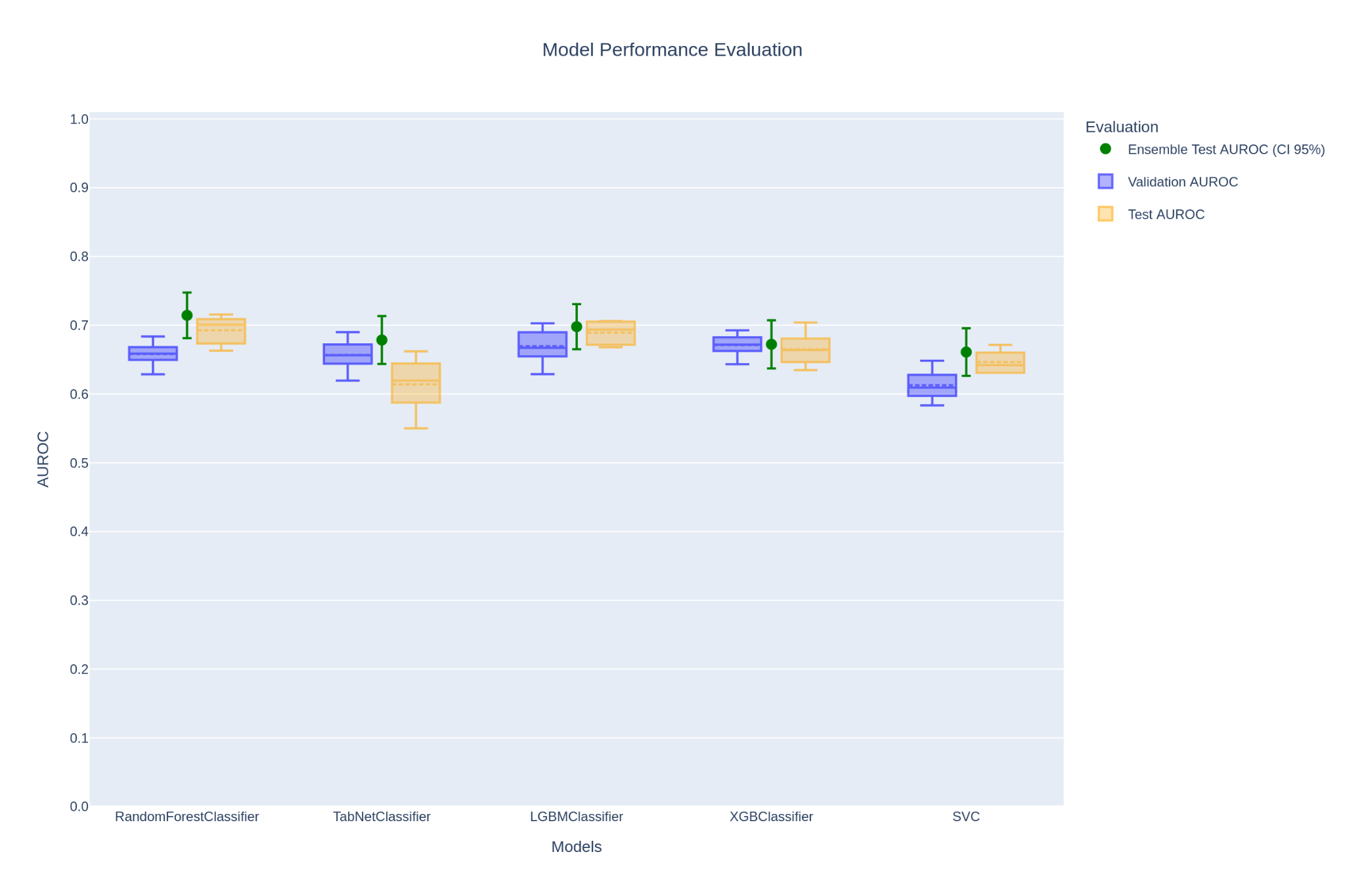


*Figure S5. Model performance for adenoma vs. CRC classification based on PyRadiomics features using the RPTK framework.*

*
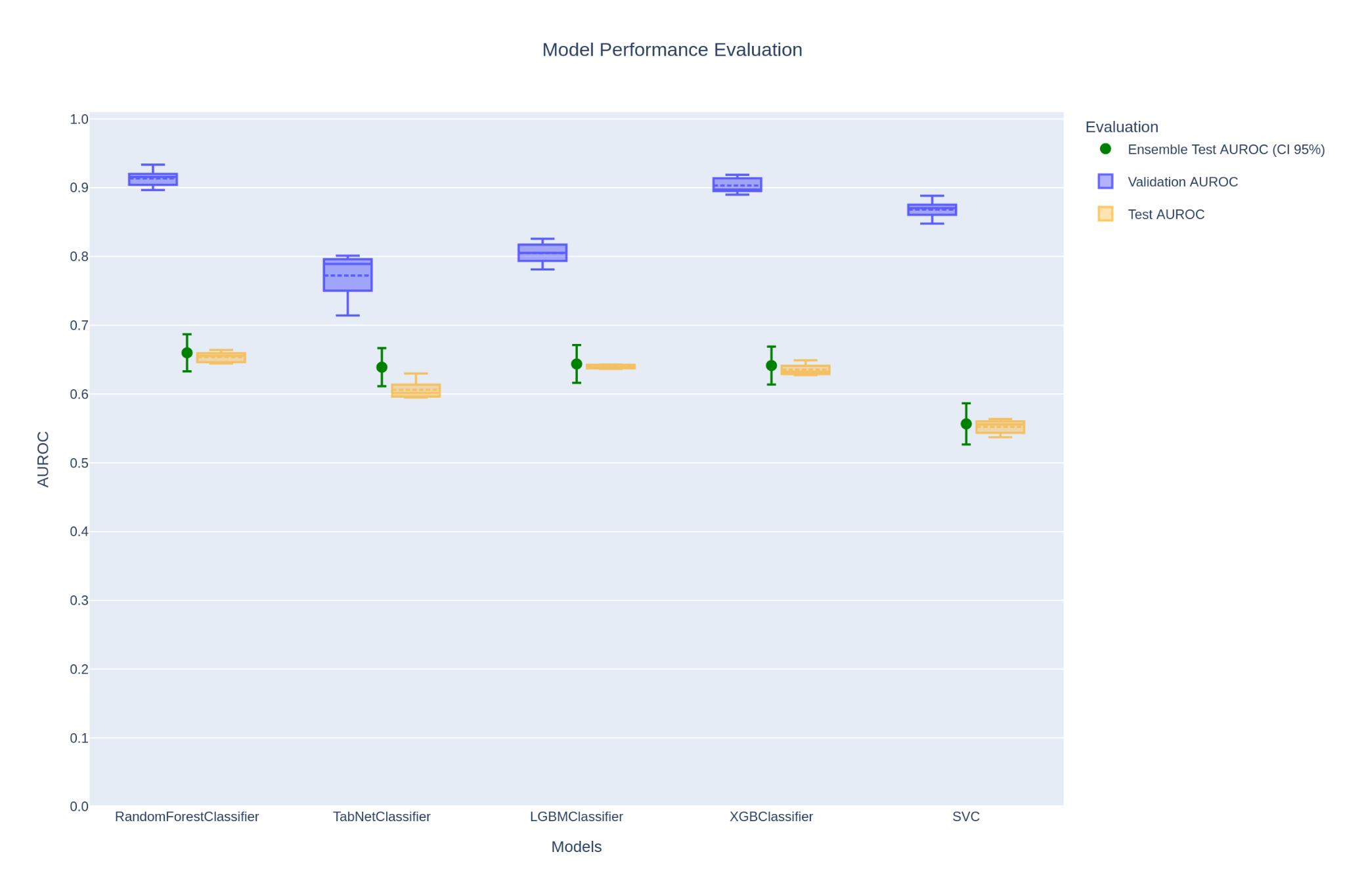
Figure S6. Model performance for CRC vs. no colorectal neoplasia classification based on PyRadiomics features using the RPTK framework.*

*
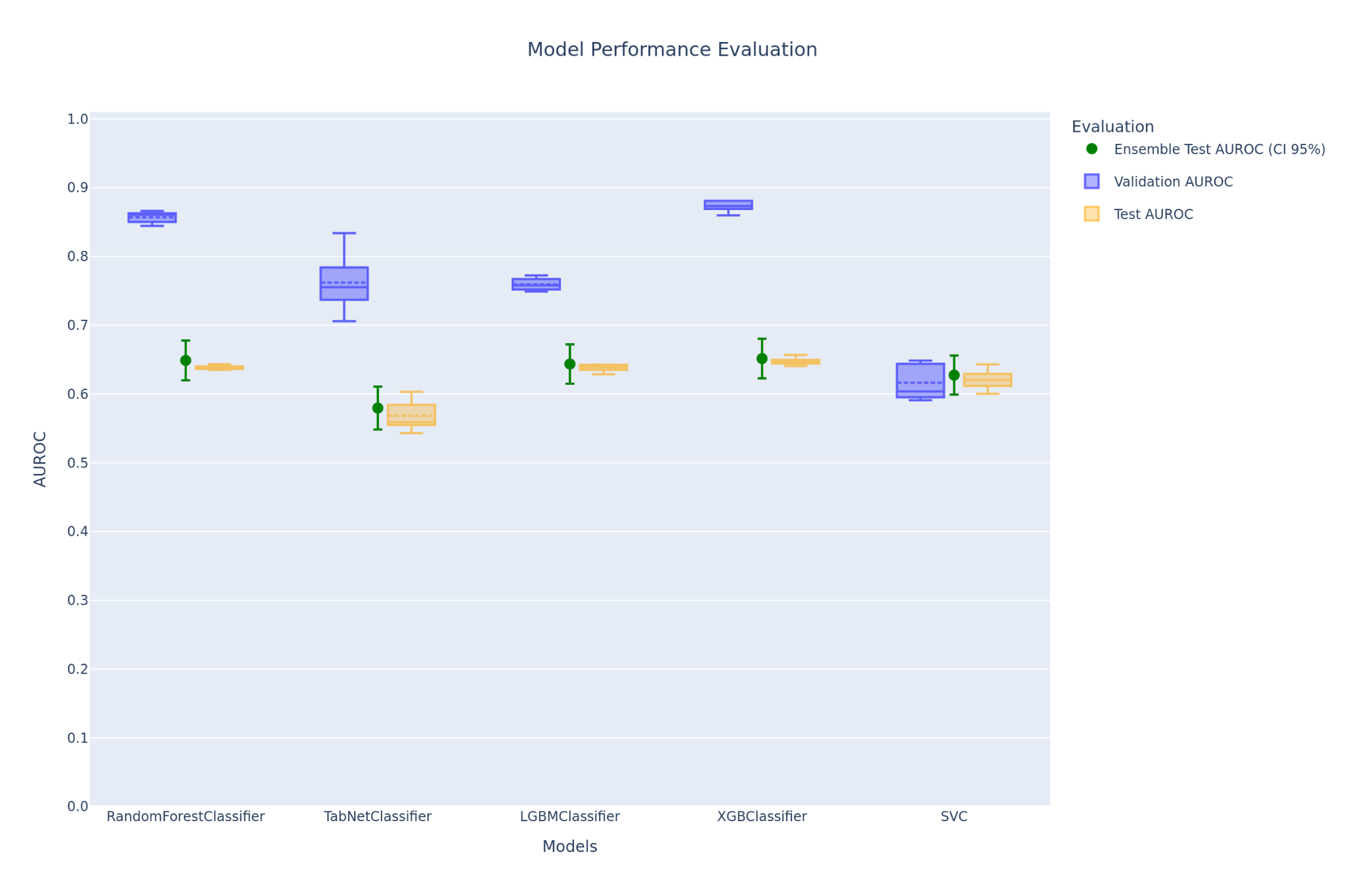
Figure S7. Model performance for adenoma vs. no colorectal neoplasia classification based on PyRadiomics features using the RPTK framework.*
